# Relationships at Work: Integrating the Perspectives of Disability Partners to Enhance a Peer Navigation Intervention

**DOI:** 10.3389/fresc.2022.876636

**Published:** 2022-07-01

**Authors:** Deana Herrman, Christina Papadimitriou, Bob Green, Andrea LeFlore, Susan Magasi

**Affiliations:** ^1^Department of Disability and Human Development, University of Illinois-Chicago, Chicago, IL, United States; ^2^Departments of Interdisciplinary Health Sciences and Sociology, Oakland University, Rochester, MI, United States; ^3^Our Peers - Empowerment and Navigational Support, University of Illinois-Chicago, Chicago, IL, United States; ^4^Department of Occupational Therapy, University of Illinois-Chicago, Chicago, IL, United States

**Keywords:** disabled person, social determinant of health, peer support intervention, community based participatory research, narrative exemplars, healthcare access, health equity, relational process

## Abstract

**Objective:**

The Our Peers-Empowerment and Navigational Support (OP-ENS) community-based participatory research study developed, implemented, and evaluated a peer navigator intervention aimed at improving health and healthcare access among Medicaid beneficiaries with disabilities. Peer navigators are community partners with physical disabilities trained to deliver structured peer support interventions that can address barriers to care. The purpose of this paper is two-fold. First, it explicates the relational work the peer navigators do with peers in delivering the intervention. Second, it illustrates how our community-based participatory approach empowered peer navigators to share their knowledge to refine the intervention.

**Methods:**

Clinical coordinator team meeting notes, critical incident documentation reports, peer navigator reflections (*n* = 20) were analyzed thematically to understand the peer navigators' relational work and intervention refinements. Following Labov's 6-stage approach to personal narratives and a collaborative writing process academic, clinical, and disability partners co-wrote descriptive exemplars to showcase these processes.

**Findings:**

Through the manualized OP-ENS intervention process, peer navigators helped peers achieve incremental successes. Peer navigators used their training and personal experiences to engage with peers and forge deep connections and relationships of trust. As a result, peers identified a wide-range of social health concerns, including poverty, social isolation, and racial and disability related discrimination that might otherwise go unaddressed. True to the principles of community-based participatory research, by fostering an equity-focused collaboration and listening to peer navigators, the project team implemented subtle but salient refinements to the intervention. Refinements included an explicit focus on social determinants of health affecting peers' health and wellbeing and supplemental trainings to help peer navigators support peers with significant mental health needs.

**Conclusion:**

The peer navigators were intentional and skilled at relationship building, thus complex elements which impact peers' health were addressed. Peer navigators were empowered to communicate their perspectives with the study team, who worked together to strengthen the intervention processes and infrastructure. This atmosphere of trust and collaboration amongst diverse stakeholders was instrumental to OP-ENS' successful implementation. Healthcare systems should consider implementing peer support interventions that are responsive to consumer input to address social determinants of health for persons with disabilities.

## Introduction

Social determinants of health are shown to have a significantly higher impact on individual and population health outcomes than does medical care (1–3). In addition to healthcare access and quality, social determinants of health are conditions in environments where people live such as employment opportunities which provide economic stability, education access and quality, accessible and affordable housing or transit in neighborhoods, and community relationships and supports which influence social and community context (3). Tackling social determinants of health as a means to reducing health disparities is a global health priority (4–7). Many countries and international agencies focused on public health are developing creative solutions to more effectively address social determinants of health among the people they serve (5, 8). A growing emphasis on person centered care as well as the strategic use of patient navigators and community health workers is yielding positive outcomes for many communities adversely impacted by social determinants of health (9–14). As incremental progress toward health equity is made, it is important to reflect on which groups are being left behind or falling through the cracks.

The one billion people worldwide experiencing some form of disability may experience exclusion to everyday activities as a result of unequal access to education, employment and disability related services, leading some to call people with disabilities (PWD) an unrecognized health disparities population (6, 15). People with disabilities face significant physical, financial, and structural barriers to health and healthcare access (16–18). Healthcare providers are often not prepared to address the comprehensive preventive, primary and specialty healthcare needs of people with disabilities (19, 20), which can lead to delayed, incomplete, and poor-quality care (21, 22). Further exacerbating the situation, many people with disabilities live at the intersection of multiple minority statuses based on race/ethnicity, gender, and socioeconomic status and are thus disproportionately disadvantaged by social determinants of health related to employment, education, economic stability, and neighborhood (23, 24). In spite of their compelling needs, PWD are largely absent from clinical, research and public health equity agendas. There is a critical need for interventions aimed at helping PWD break down barriers to health and healthcare access.

Peer health navigation is a type of peer support intervention designed to help people break down barriers to healthcare (25). Peer health navigators (PHN) are persons who share similar lived experiences with community members they serve and are trained to provide individualized emotional, informational, and instrumental support to peers to address health and healthcare needs (26, 27). To be effective in their roles, PHN and peers must create trusting, working relationships (28–30). In spite of the importance of rapport building as part of the peer health navigation process, there is a paucity of information about how this important relational skill is actually performed in action, in general, and in the context of addressing the healthcare needs of people with disabilities, in particular.

This paper explicates the complexities of rapport building within the context of the OP-ENS (**O**ur **P**eers—**E**mpowerment and **N**avigational **S**upport) peer health navigation study. Additionally, this paper illustrates the importance of collaborating with people with disabilities as integral members in the development, implementation, and evaluation of health interventions by sharing the ways in which the OP-ENS project fully realized a community-based participatory approach. Specifically, we will detail how we refined the intervention based upon PHN's experiences from working with peers. We use two data-driven exemplars to describe the nuanced ways PHN engaged with peers to build rapport and effectively address healthcare access needs as well as underlying social determinants of health, such as structural racism, poverty, and social isolation. Understanding these relational processes allow us to see how PHN deliver person-centered services to address peers' health and healthcare needs.

## OP-ENS Community Partnership, Intervention Development, and Implementation

### OP-ENS Team and Community Partnership

OP-ENS was developed *via* a community-based participatory research (CBPR) partnership between academic disability researchers, a Medicaid managed care organization, and disability community partners from the health policy team at a local Center for Independent Living between 2014 and 2020. Centers for Independent Living are community-based organizations run by and for people with disabilities with an emphasis on disability rights, advocacy and empowerment. The principal investigator of the project (SM) had been working closely with the local center for independent living for a decade before OP-ENS was developed, which forged strong and foundational partnerships among the academy and disability community. CBPR is a method to integrate the experiential knowledge of PWD and respond to the disability communities demands to control how healthcare and research are structured to address their needs. The goal of CBPR is to create meaningful partnerships between community members and researchers to address health needs (31, 32). CBPR prioritizes equitable collaboration by recognizing the unique strengths of the team members, throughout all stages of the research process to improve health and participation of a community (31, 32). OP-ENS is an example of including the expertise, lived experiences and perspectives of PWD throughout the research process (33, 34).

### OP-ENS Intervention Theoretical Grounding

OP-ENS is a 12-month long manualized peer health navigator intervention wherein a cohort of trained persons with physical disabilities work directly with peers with physical disabilities using a structured process of goal setting, barrier identification and asset mapping, and action planning to help their peers achieve their health management and healthcare access goals. Exclusion criteria included people with severe and persistent mental illness as the training needs to adequately support this community were deemed to be beyond what could reasonably be expected of the lay PHN. OP-ENS is grounded in self-efficacy and stress and coping theories (25). Specifically, PHN help peers build a repertoire of success by supporting and role modeling active self-management while also providing on-going, dependable social support over the 12-month intervention period. PHN met with peers at least monthly, either in-person or by phone. Frequency and duration of these sessions varied based on peer-identified needs. The duration of the intervention and the blend of self-efficacy and social support theoretical groundings acknowledge the fluctuating nature of the disability experience that can vacillate between periods of relative stability and periods of uncertainty due to shifting health and social service needs. OP-ENS takes a broad view of health and healthcare recognizing that transportation, housing and food security are as important to people's abilities to manage their health as are the in-clinic experiences (33, 35, 36).

### PHN Training

OP-ENS PHN complete 40-h of initial training. This initial OP-ENS training was developed through a collaborative team process. Partners from the participating entities worked together to create an overall curriculum map that identified the knowledge and skills needed to deliver the OP-ENS intervention. Individual partnership teams then took the lead on developing content modules within their areas of expertise. Specifically, disability community partners were responsible for creating modules on the history and legacy of the disability rights movement, disability competence and etiquette, and an overview of barriers to healthcare that confront people with disabilities. Partners from the Medicaid managed care organization (MCO) developed content related to understanding healthcare delivery and coverage, the MCO's processes of care coordination and service provision, and patient protection and privacy issues. The academic team, many of whom have clinical backgrounds in occupational therapy, created content on the intervention process (rapport building, barrier identification and asset mapping, goal setting, and action planning) and requisite skills such as communication, active listening, documentation. Consistent with best-practices for PHN training, the initial OP-ENS training included content on role delineation, boundaries, and when to make referrals. A master trainer in motivational interviewing was contracted to provide specialized skills training. All training modules were reviewed and revised to ensure that they adhered to principles of universal design for learning, plain language and included opportunities for application and active learning. The training was delivered by diverse members of the collaborative team over a 4-week period and occurred at the center for independent living. Ten trainees were recruited from community-based networks and completed a rigorous screening and interview process. All 10 trainees successfully completed the training course as verified by daily knowledge checks and practicum with members of the disability community. Ultimately, nine of the trainees were hired to serve as peer health navigators for the implementation phase of the study. For more detail on how PHN were chosen and trained, see Magasi et al. (33).

### OP-ENS Transition to Implementation

As the project transitioned from the development phase to implementation, the roles of the collaborative partners shifted in ways that were both intentional and unanticipated. The center for independent living provided community outreach, on-going project oversight and consultation on implementation, as well as a vital links to community-based resources, advocacy and support. The MCO served as the referral source for the initial phases of implementation and strong efforts were made to recruit from their member-base in collaboration with the professional MCO case management team. Unfortunately, due to political changes in the state government, the MCO lost its funding and went out of business about a year into the intervention phase of the study. As a result, the project lost its “clinical anchor” and access to medical documentation as part of the screening and enrollment processes. The project team shifted to community-based recruitment and conducted extensive community outreach through a variety of community-based organizations and clinical entities who serve the disability community.

### Implementation

PHN worked directly with community-dwelling peers with physical disabilities to deliver the OP-ENS intervention according to the manualized intervention protocol. As OP-ENS was implemented, the PHN were integrated into the project team as both interventionists and key partners. PHN were encouraged to share their insights from working with peers with the project team. This enabled the project team to implement enhancements to the intervention and supplemental trainings to increase the PHN's ability to effectively support their peers (37).

The PHN team met for bi-weekly team meetings, which were facilitated by the clinical coordinator, experienced occupational therapists and doctoral students at the time of the study. The team meetings emerged as a highly collaborative workspace where PHN were empowered to share their experiences of responding to the participants needs and identify ways that the project infrastructure could flexibly adapt to address those needs while still maintaining fidelity to the OP-ENS process at the heart of the intervention (33, 37). Fidelity was monitored by a research team member not associated with OP-ENS implementation by reviewing monthly contact log and PHN documentation to ensure adherence to both the recommended OP-ENS process and dosage.

## Methods

Data for this paper came from multiple sources. During the OP-ENS intervention, clinical coordinators documented peer navigator team meetings, wrote critical incident reports, and reflections on their supervision of PHN. At the same time, peer navigators documented their reflections of the OP-ENS process including about their interactions with peers, team meetings, and supervision. The principal investigators of the study routinely reviewed these reports and became aware that they were rich with information about how PHN worked with the OP-ENS intervention procedures, how they implemented the intervention with each peer, and the facilitators or challenges they experienced with these processes. As part of the OP-ENS program, we used these reports and reflections to respond to PHN needs, such as by offering professional development opportunities and trainings (for example, mental health first aid training), focus supervision on particular topics of concern (racism, extreme poverty), and provide increased opportunities for peer support.

Based on our commitment to equitable partnerships and the recognition of the value of stakeholder engagement, we invited the clinical and PHN teams to jointly craft written exemplar narratives based on their work with peers to showcase the work that takes place among PHN and peers. Two PHN were available and interested to participate in writing exemplars. We therefore engaged in a collaborative writing process, using Labov's 6-stage approach to writing effective narratives (38). The writing team involved 2 PHN, one clinical coordinator, the academic principal investigators, and undergraduate and graduate students.

We began the writing process by de-identifying clinical notes and reflections and copying them into Word files (*n* = 20). The PHN and clinical coordinator were responsible for assuring the accuracy of the information presented in the working document and added detail or adjusted information as needed. The academic partner (second author) edited the draft to apply Labov's approach and ensure that key aspects of the peers' narratives were communicated in engaging and organized ways. Labov suggests that a compelling story starts with a short summary (step 1), usually one or two sentences, that lets the reader know in general terms what the topic of the story is. Then he suggests to add orientation and background detail (step 2) to orient the reader to when and where the story takes place, and then follow with writing that builds suspense (step 3). A statement of evaluation of why the story is worth telling (step 4) may come next followed by a resolution (step 5) of suspense. Finally, ending the story (step 6) with a statement that leaves the reader with a feeling of satisfaction and completeness.

The exemplars that we share in this paper have been revised 7 times and reflect the collaborative writing of the whole team. During our writing and revision, the writing team met every 2 weeks to discuss the edits and reach simple consensus. Given team members' differing writing styles, Labov's approach afforded us a writing structure that we all agreed on and felt comfortable working with.

Ethics approval was received at all participating sites (University of Illinois-Chicago, Protocol #2015-1207).

### Data Analysis

Analysis began with multiple readings of the aforementioned data and the selection of narrative exemplars that the team wanted to focus on. The selection was initiated by the PHN who brought several narratives to the team for consideration. The PHN selected examples that exemplified how the processes of rapport and trust building were used to support peers in achieving pressing short-term goals while enabling the long-term exploration of pervasive needs created by deep seated issues related to social determinants of health. Two PHN, Bob and Ryann, participated in the co-creation and interpretation of the exemplars. In close collaboration with Bob and Ryann, the second author led the iterative process of writing, editing and interpreting them. All members of the authorship team provided feedback and participated in the analysis presented in this paper. The iterative, collaborative process is a common strategy in qualitative research designs (39). We decided to include two narrative examples in this paper due to space limitations with manuscripts.

We engaged in thematic analysis of the data (40). We immersed ourselves in the data and asked ourselves “what are the narratives telling us regarding the relational processes of peer navigation?” During analysis, team members expressed a variety of life experiences, disciplinary backgrounds, and positions only some of which overlapped. This made for vibrant discussions in which we unpacked our taken for granted assumptions about the emerging themes. We shared drafts of our writing and wrote comments in the written documents as well as during meetings. The second author kept and shared notes with the writing team *via* email and in shared electronic drives. The writing team worked on writing drafts together in video-conferencing calls where we could read and comment on documents together.

The first exemplar that features Charlie brought up the themes of systemic racism and challenges of interacting with healthcare bureaucracies. The second exemplar that features Sarah brought up themes of isolation and trauma. Both exemplars share in common the relational work that peers engaged in with participants as well as some of the refinements made in the OP-ENS intervention.

Within our CBPR conceptual approach to our overall work, we engaged in critical reflection by exploring our own assumptions and understandings regarding the themes that were emerging, and then engaged in reflexivity which involved acknowledging our own positioning to understand the limitations of our own perspectives and better appreciate those of others (37).

## Findings

### Narrative Exemplar 1—Uncovering the Impact of Systemic Racism

Bob is a 50-year-old Black man who lives independently in a large Midwestern city. In 2014, he had a hemorrhagic stroke and experienced aphasia, impaired mobility, left side hemiplegia and neglect. Pre-stroke, he was part of research teams in Neonatology and Biochemistry. He joined the OP-ENS team as a peer health navigator (PHN) in 2015. He worked with Charlie (pseudonym) who is a 42-year-old Black man and lives in an under resourced community in a large Midwestern city.

At 19, Charlie experienced a gunshot wound which left him with paraplegia. Charlie used his manual wheelchair for transportation and fitness; thus, his wheelchair was vital to both his health and community participation. Heavy use of his chair on city streets caused its misalignment and led to the development of pressure sores on one leg. Charlie hoped OP-ENS could assist in repairing his wheelchair by a seating company with whom he had been communicating without success for 3 months.

Charlie felt that discrimination against his neighborhood was leading to scheduling failures. He had negative past experiences with the healthcare system including unacceptable wait times, inattentive physicians, and dismissiveness and disregard of his health complaints. Charlie stopped trying to schedule the repair and continued to use his misaligned chair for transportation, leading to worsening health.

During the first 6 months of the OP-ENS intervention, Bob and Charlie developed a trusting relationship, which allowed them to learn more about each other and share common experiences. They engaged in difficult and emotional conversations about racism and perceived racism. During this time, Charlie expressed that his major health concern was his pressure sore and his reticence to seek medical care due to his negative experiences with healthcare providers. Eventually, with Bob's support, Charlie arranged a 3-way call between the repair company, PHN, and himself in which he was able to resolve the scheduling problem. His wheelchair was repaired within 5 days of the phone call. When Charlie's pressure sores became infected, Charlie sought medical treatment in a timely fashion without prompting from the PHN.

#### Synthesis

Charlie initially stated he had “no health concerns, other than I need my chair fixed and I'm getting old and not healing like I used to.” It took months of relationship building to learn about his pressure sore. At the beginning of their relationship Charlie gave Bob the moniker “schoolboy” because he did not believe a person whose socioeconomic and educational background was so different from his own could understand his daily challenges. As part of his training and the OP-ENS project infrastructure, Bob appreciated that it was his responsibility to initiate and maintain the relationship. Building rapport is a relational practice that creates emotional connections and trust through active listening, strategic intentional sharing, and dependability. Debriefing and team-based problem solving in the bi-weekly PHN team meetings as well as extensive conversations with and between the clinical coordinator and project leaders allowed Bob to process his reactions to Charlie's behavior, work out how to establish rapport and trust with him, and receive additional supports on how to acknowledge Charlie's experiences while also serving his immediate healthcare needs. The project team also recognized that while OP-ENS is founded on practices of goal setting and action planning, it was important to give the PHN freedom to engage in prolonged relationship building. The ability to collaborate on deeply rooted problems was predicated on trust and relationship.

During their monthly phone check-ins, Bob and Charlie identified a mutual passion for fitness and sports. Their conversations about engaging in physical fitness as disabled men helped them see their commonalities and served as the foundation for rapport. Soon after, Charlie told Bob about his pressure sore and his reluctance to seek medical care because of previous negative racial attitudes toward him by healthcare staff. He believed that his wheelchair repair delays were due to race-based discrimination against the community where he lived. The two began to discuss racism in general, how to manage discrimination without being self-destructive, and how to manage getting one's health goals met regardless of negative experiences with healthcare providers. The intersection of racism and disability was familiar to Bob. As a Black man, Bob understood that Charlie's lens of negatively racialized experiences impacted his ability to effectively manage his health and access healthcare. Due to their shared understanding, and Bob's OP-ENS training, Bob was able to “meet Charlie where he was at” and engage him in frank discussions to implement strategies that would allow him to navigate the healthcare system and get his needs met. For example, Bob helped Charlie recognize that talking angrily to customer service representatives and openly accusing them of racism, while grounded in his experience, was ultimately counter-productive to his goal of getting his wheelchair fixed. They worked together to come up with an alternate approach that allowed Charlie to effectively communicate his needs and get the results he sought. Staying focused on Charlie's expressed needs allowed for frank discussions over time about systemic racism and stigmatizing experiences, which validated Charlie's experiences of discrimination.

Please see Supplementary Video developed from this exemplar (41). The video shows how Bob and Charlie's relationship developed and how the two tackled barriers Charlie faced in getting his health needs met.

### Narrative Exemplar 2—Tackling Social Isolation and a History of Trauma

Ryann is a 33-year-old Black woman, who lives with her young child. She experienced a traumatic brain injury in 2006 due to a gunshot wound months before her high school graduation. She participated in rehabilitation services after her injury and with the support of her family, transitioned into community living. At the time of the study, she was in the process of completing her associate degree in mortuary science at a local community college. She became a PHN with OP-ENS in 2015. Ryann and Bob worked together with Sarah (pseudonym).

Sarah was a White middle-aged woman who lived alone in an old trailer in a rural community. She had multiple experiences of domestic violence, at the hands of her father who was an active alcoholic and many of her five ex-husbands. She had six children, but was estranged by all of them except her eldest son, who had been recently diagnosed with early onset Alzheimer's disease. Her support system included her eldest son, a neighbor who occasionally helped with house repairs, and her dog.

Sarah showed competency navigating various aspects of the healthcare systems but her relationships with her doctors were contentious. She indicated that her “doctors did not listen” to her. She refused their advice on many instances. For example, when her primary care physician suggested that her obesity was the result of her depression and urged her to see a psychologist, she adamantly refused. Additionally, she experienced transportation challenges including unreliable services and inability to be transported safely. Sarah asked the PHN for support with her Personal Attendants (PA) because she had trouble keeping them.

Sarah worked with two PHN, Bob and Ryann. OP-ENS calls for a minimum of monthly phone meetings between PHN and peers. As the rapport between Ryann and Sarah developed, Sarah would call the office 2–3 times a week. Ryann recognized that Sarah needed greater support and that Sarah's history of trauma and abandonment made it important for Ryann to demonstrate that she was a stable presence and trusted person in Sarah's life. The research and clinical teams discussed the need to adjust our approach to serve Sarah's needs while maintaining intervention fidelity. Ryann, Bob and the clinical coordinator decided that Ryann could increase the frequency of her phone contacts, while also being careful to establish healthy boundaries and diligently watched for signs of needing to refer Sarah to professional mental health services.

Sarah and Ryann developed a close bond, talking weekly, and discussed Sarah's stressful relationship with her daughter. Sarah began to reconcile her relationship with her daughter which allowed her to connect with her grandchildren. When Sarah stated she was determined to lose weight so that she could “use a walker to shop with her daughter and grandchildren”, Ryann offered weight loss resources to her.

Soon after, Sarah identified that the demands of living alone were too much for her and requested help finding assisted living accommodations. While OP-ENS is grounded in the belief that people with disabilities have the right to live in the least restrictive environment, Ryann and Bob had come to appreciate the profound impact that social isolation had on Sarah's health and wellbeing and supported her choice. A few weeks after Sarah settled into her new environment, Bob and Ryann visited her. Sarah was so happy to have them visit her; it was clear that her attitude had become positive and that she felt a part of the facility's community. She introduced us to therapists and other residents as her “friends”. She showed off standing between the parallel bars and was taking steps with her walker. The transformation was remarkable. Sadly, insurance issues forced her to leave the assisted living facility and after trying for a month to reach her, her daughter informed us that Sarah had passed.

In response to the PHN experiences in working with Sarah and other peers with significant histories of trauma and mental health concerns, the project team invested resources to provide supplemental trainings in mental health first aid, motivational interviewing, and an introduction to trauma-informed care.

#### Synthesis

Sarah built rapport with both Ryann and Bob, but gravitated to Ryann because of their shared experiences as women with disabilities in a healthcare system that is difficult to navigate and where providers do not always listen to their needs. Ryann was an empathetic listener, who also shared her own experiences with medical challenges related to her disability, urinary incontinence, as well as about her experiences of trauma and loss. As Ryann and Sarah deepened their connection, Sarah felt safe enough to explore additional concerns, like how her loneliness and isolation affected her mental, physical and social health. In her words, “my loneliness is hell, I don't know how much more I can take”. Ryann was “there for” Sarah; listening, sharing, and overall validating Sarah's feelings.

While OP-ENS is founded on tenants of the disability rights and independent living movement that advocates for community-based living for people with disabilities and many of the OP-ENS PHN personal narratives include fighting against nursing home placement for themselves and members of the disability community, PHN demonstrated an ability to not let these positions cloud their ability to support Sarah's self-identified needs. The team empowered Sarah to make the choice that was right for her: a choice that gave her access to regular social interactions, scheduled activities, assured meals, and timely medical attention. The PHN shifted between their PHN role, their disability identity, their own experiences in the healthcare system and society, as well as perceptions about placement to truly center support around Sarah's needs. The PHN supported Sarah's health needs on many levels: practical (reliable PA support), emotional (loneliness), social (isolation, supportive relationships), medical (obesity, depression, mobility), mental health (depression, loneliness, social support), structural (assisting living, insurance coverage, healthcare access), financial (living in poverty, insurance coverage). This case highlights the importance of “being there” by listening to peers' needs, validating their experiences, and working together to create an action plan that best fits them.

## Discussion

**Figure 1:**
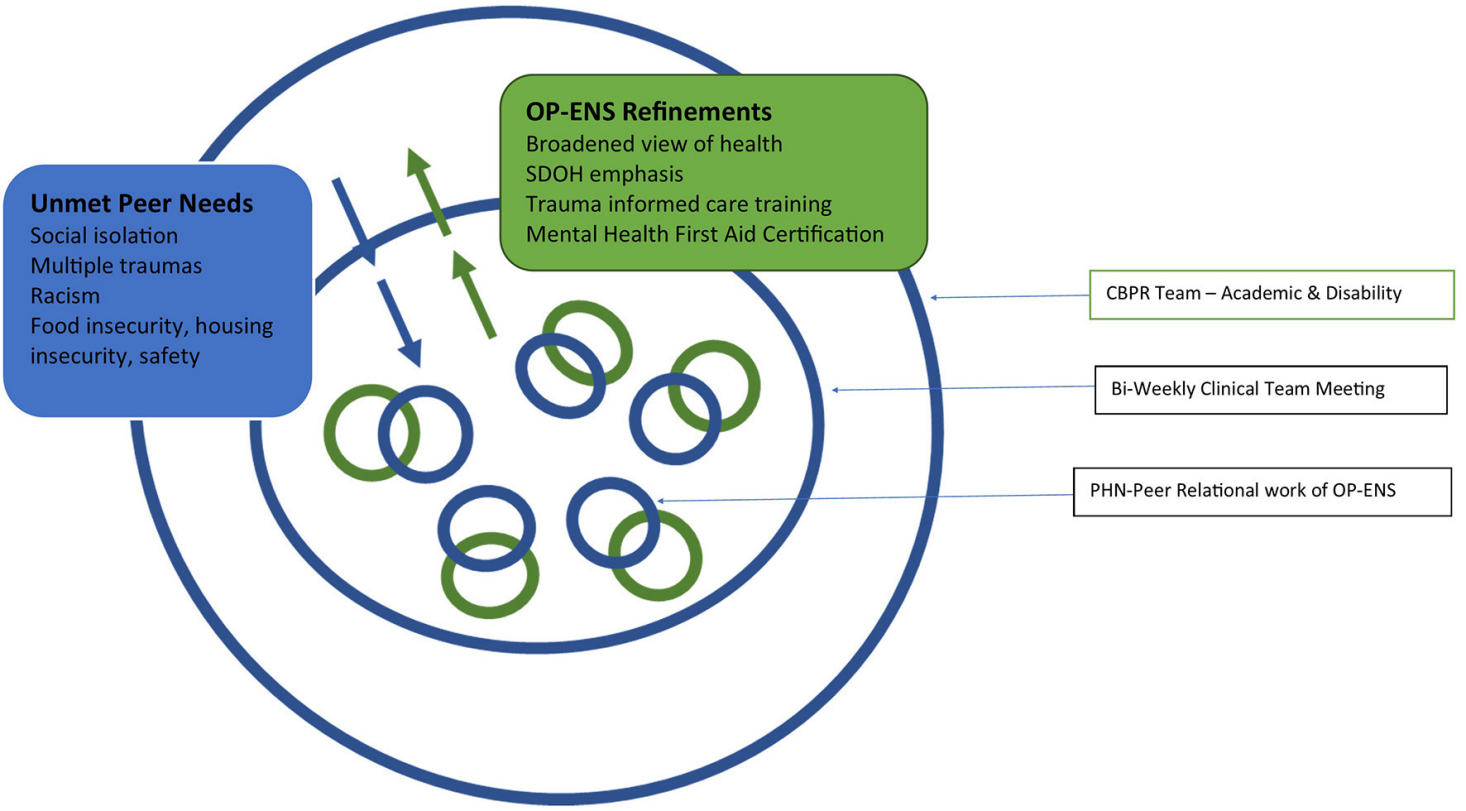
Centering communications and creating refinements in the OP-ENS intervention.

These two exemplars illustrate the complex and multifactorial issues that were impacting the health, function, and quality of life of the people OP-ENS was aimed to support. Rapport building is typically conceptualized as a core component of peer support interventions and indeed the PHN built trusting relationships with peers to deliver the intervention and meet the peers' needs. As PHN brought these experiences back to the whole team for discussion and collaborative problem solving, opportunities arose to further strengthen the project team's infrastructure to support the PHN and by extension the people served. Figure 1 represents how multiple bi-directional (i.e., PHN-peer and PHN-OP-ENS team members) communications centered in the PHN-peer relational work led to refinements in the OP-ENS intervention, PHN training, and project structure.

The OP-ENs intervention was designed with a relatively broad conceptualization of health. Continually learning about peers' unmet needs and priorities during the back and forth between PHN, the clinical coordinator, and the project leadership challenged the team to further broaden those definitions and fully embrace perspectives aligned within the framework of the social determinants of health (5). This included increasing frank discussions about structural issues, like systemic racism, community violence, and gender-based trauma. The OP-ENS team's efforts to create an environment of respect, trust, and collaboration between all members of the team regardless of role was essential to creating a safe collaborative space where these issues could be brought into the open and nimbly integrated into the intervention to strengthen its relevance and responsiveness to the people we served. Specifically, while our exclusion criteria were designed to screen out people with severe and persistent mental illness, the PHN quickly recognized that many peers had histories of trauma and unresolved mental health concerns. The PHN and project team agreed that addressing these mental health needs was an important area for continuing training and education to enable the PHN to adequately support their peers. Therefore, drawing on their clinical background in occupational therapy, the clinical coordinator introduced the concepts of trauma informed care and how they can be used to support people we serve. The team also contracted with an external agency to train the PHN in mental health first aid. All PHN became certified in Mental Health First Aid and built both their knowledge and self-efficacy around how to support people with mental health concerns, while maintaining professional boundaries and recognizing the limits of their expertise.

These exemplars reveal the PHN's abilities to provide a uniquely responsive form of support to navigate healthcare systems. This support not only acknowledges the impact that social determinants of health have on the people they serve, but effectively centers the disability experience to help identify and begin to address the impact of deep-seated issues, like systemic discrimination and social trauma (23, 42, 43). PHN were able to center not only the individual peer's wants and needs, they were also able to center the disability experience on relational and shared lived experiences (44, 45). For example, PHN know about healthcare systems barriers, often have lived the intersection of racism, sexism and ableism, and have been trained to acknowledge those realities in order to center on understanding peers' wants and needs, while also co-creating achievable health goals. PHN's engagement with OP-ENS teams and OP-ENS participants fostered the development of trusting relationships and enabled the PHN to refine the intervention by addressing the need of the peers. PHN built rapport over their shared experiences and interests, both physical in nature as in Bob and Charlie's love of fitness, and medical challenges as in Ryann and Sarah's discussions of urinary incontinence and physical therapy regiments. These connections provided a foundation for engaging in goal setting and action planning components of the OP-ENS intervention to address discreet health needs. Success begets success and as the two worked together to find solutions to pressing needs, the relationship deepened, and trust was established. The peers began to recognize that the PHN was someone who could be trusted to understand their needs, provide tangible support, and “be there” over time when needed.

It is within this foundation of trust that peers felt safe and empowered to raise deeper and potentially more pervasive concerns, such as the impact that structural racism had on Charlie's willingness to seek care or how Sarah's history of trauma and abuse contributed to her social isolation and physical and mental health challenges.

Also striking were the PHN skills at finding and building connection. An exact match in clinical and demographic characteristics was simultaneously not necessary and not enough. For example, Bob had to break through Charlie's perceptions that differences in socioeconomic status and education rendered Bob incapable of understanding his experience, even though they were both Black men with disabilities. On paper, it seemed as though Ryann, a young, big city Black woman with a supportive family had little in common with a down state, middle-aged White woman experiencing social isolation. Yet both were able to form supportive relationships by centering their shared experiences while maintaining focus on the peers' personal goals and needs. These skills are common components of peer support interventions (9, 29). PHN may shift between their role as peer supporters, their own experiences and values (46) and their training to carry forward peers' needs and “meet them where they are” (29). This relational skill allows PHN to deliver tailored supports with respect. PHN were trained in these skills and received continuous support as they applied them with specific peers during the OP-ENS intervention. The relational support among PHN and among PHN, the clinical coordinator, and larger OP-ENS team should be emphasized since those support structures allowed for open communication of needs and supports, increased our responsiveness to peer and PHN needs, and fostered collaborative learning among all OP-ENS team members (33, 34).

### Implications

We suggest that people with disabilities should be included in healthcare research interventions and healthcare delivery teams. Engaging the disability community as active stakeholders in healthcare research interventions can allow disabled people, healthcare providers and researchers to try to solve larger issues related to social determinants of health by navigating the social, economic and political environments that impact the health of individuals and the larger community. Peer supporters are one way to engage with people with disabilities and be inclusive of their lived experiences. A structured yet flexible framework which allows open communication of needs and supports and collaborative process allows each stakeholder to draw upon their areas of expertise to inform interventions to benefit the recipients of the intervention. Ensuring that time for training and flexibility for meetings and communication may be useful elements to integrate when designing peer support interventions. The OP-ENS team's commitment to listening and learning together strengthened the PHN training as well as the implementation and responsiveness of the intervention. This flexible approach to support the PHN's and peers' emergent needs while maintaining fidelity to the evidence- and theory-informed components of the intervention can serve as a model to teams seeking to develop and implement peer support interventions (47). Furthermore, there is increasing recognition of the prevalence of unresolved trauma and mental health concerns in the general population as well as amongst people with disabilities. Intervention developers and implementors should be prepared to address co-occurring mental health concerns, including the provision of on-going support and making appropriate referrals when the participant needs exceed the PHN's training and capacity to support.

### Limitations

This secondary analysis of existing study and intervention documentation materials has several limitations. First is the use of clinical intervention notes (both PHN and clinical coordinator notes) that were designed for documentation and tracking of the intervention. They were not intended to serve as qualitative data and therefore may not contain the level of first-person narrative that would afford us to do rigorous qualitative analysis. The voice of the peer is not directly recorded. Future research should seek to engage with peers to explore their experiences for the relational aspects of peer support and peer health navigator interventions. We center our explorations on a limited set of data from two study exemplars. We acknowledge that these exemplars may not be representative of all encounters and findings are not generalizable. We do, however, assert that the in-depth exploration of two powerful exemplars sheds light on the broader potential of centering disability experiences as to address social determinants of health at the intersection of disability, race, trauma, and poverty. Further examining how PHN enact person-centeredness is an avenue for additional research. Similarly, the role of a PHN as a valued member of an interdisciplinary healthcare team should be further explored. It is unclear how the loss of the Medicaid managed care organization as a referral source influenced the peer-identified needs and co-occurrence of mental health concerns. It should be noted, however, that many of the issues, including the social isolation, unresolved trauma and the recognition of the need for an expanded view of health were identified early in the project's life-cycle while the Medicaid managed care organization was still in operation and should be integrated into the training and intervention planning for all peer support interventions.

## Conclusion

While delivering OP-ENS, a peer support intervention, the PHN abilities to flexibly negotiate challenging emotional, experiential and social determinant of health factors allowed them to establish trusting relationships with peers. Collaboratively the PHN and peers were able to identify and address health immediate concerns while also acknowledging the real impact of contributing factors like systemic racism, ableism and extreme social deprivation resulting (in part) from interpersonal trauma. The power of rapport building amplifies the peer-centeredness of the intervention as the PHN engage with topics not always shared with medical professionals and fully support peers in a manner that centers on the peers' experiences and needs. As a CBPR intervention, OP-ENS was explicit that the PHN were integral members of the team, not just as interventionists but as collaborators and contributors to the intervention's community relevance and successful implementation. By learning from and with the PHN about their experiences and the needs of their peers, the project team was able to introduce enhancements to the intervention process, the PHN training, and the program infrastructure while maintaining intervention fidelity.

## Data Availability Statement

The raw data supporting the conclusions of this article will be made available by the authors, without undue reservation.

## Ethics Statement

The studies involving human participants were reviewed and approved by University of Illinois-Chicago, Protocol 151#2015-1207. The patients/participants provided their written informed consent to participate in this study. Written informed consent was obtained from the individual(s) for the publication of any potentially identifiable images or data included in this article.

## Author Contributions

SM and CP contributed to conception and design of study. SM, CP, DH, and AL contributed to manuscript ideas and organization. BG, CP, and SM participated in the co-creation and interpretation of the exemplars. CP edited the drafts of exemplars. SM, CP, and DH wrote sections of the manuscript. All authors contributed to the manuscript revisions, read, and approved the submitted version.

## Funding

This work was supported with a grants from the National Institute on Disability, Independent Living and Rehabilitation Research, 56890RT5027 and 90RTCP0005-01-00.

## Conflict of Interest

The only potential conflicts of interest are related to Grant funding for SM and CP, and honoraria from the grant while working on the study for BG.

## Publisher's Note

All claims expressed in this article are solely those of the authors and do not necessarily represent those of their affiliated organizations, or those of the publisher, the editors and the reviewers. Any product that may be evaluated in this article, or claim that may be made by its manufacturer, is not guaranteed or endorsed by the publisher.
